# Detection and Characterization of Alongshan Virus in Ticks and Tick Saliva from Lower Saxony, Germany with Serological Evidence for Viral Transmission to Game and Domestic Animals

**DOI:** 10.3390/microorganisms11030543

**Published:** 2023-02-21

**Authors:** Cara Leonie Ebert, Lars Söder, Mareike Kubinski, Julien Glanz, Eva Gregersen, Katrin Dümmer, Domenic Grund, Ann-Sophie Wöhler, Laura Könenkamp, Katrin Liebig, Steffen Knoll, Fanny Hellhammer, Anna-Katharina Topp, Paul Becher, Andrea Springer, Christina Strube, Uschi Nagel-Kohl, Marcel Nordhoff, Imke Steffen, Benjamin Ulrich Bauer, Martin Ganter, Karsten Feige, Stefanie C. Becker, Mathias Boelke

**Affiliations:** 1 Institute for Parasitology, Centre for Infection Medicine, University of Veterinary Medicine Hannover, Buenteweg 17, 30559 Hanover, Germany; 2 Research Center for Emerging Infections and Zoonoses, Buenteweg 17, 30559 Hanover, Germany; 3 Institute of Virology, Department of Infectious Diseases, University of Veterinary Medicine Hannover, Buenteweg 17, 30559 Hanover, Germany; 4 Institute for Biochemistry, University of Veterinary Medicine Hannover, Buenteweg 17, 30559 Hanover, Germany; 5 Lower Saxony State Office for Consumer Protection and Food Safety (LAVES), Food and Veterinary Institute Braunschweig/Hannover, Eintrachtweg 17, 30173 Hanover, Germany; 6 Lower Saxony State Office for Consumer Protection and Food Safety (LAVES), Food and Veterinary Institute Oldenburg, Philosophenweg 38, 26121 Oldenburg, Germany; 7 Clinic for Swine and Small Ruminants, University of Veterinary Medicine Hannover, Bischofsholer Damm 15, 30173 Hanover, Germany; 8 Clinic for Horses, University of Veterinary Medicine Hannover, Buenteweg 9, 30559 Hanover, Germany

**Keywords:** Jingmenviruses, Alongshan virus, ticks, LIPS assay, artificial infection, Lower Saxony

## Abstract

The newly discovered group of Jingmenviruses has been shown to infect a wide range of hosts and has been associated with febrile illness in humans. During a survey for Jingmenviruses in ticks from Lower Saxony, Germany, Alongshan virus (ALSV) was identified in *Ixodes* spp. ticks. Additional virus screenings revealed the presence of ALSV in the bodies and saliva of ticks collected at several locations in Lower Saxony. Vector competence studies that included *Ixodes ricinus* and *Dermacentor reticulatus* validated the replication of ALSV within those tick species. In vitro feeding experiments with ALSV-injected *Ixodes ricinus* demonstrated effective viral transmission during blood feeding. To evaluate the potential viral transmission during a natural blood meal, sera from wild game and domestic animals were investigated. One serum sample from a red deer was found to be positive for ALSV RNA, while serological screenings in game and domestic animals revealed the presence of ALSV-specific antibodies at different locations in Lower Saxony. Overall, those results demonstrate the broad distribution of ALSV in ticks in Lower Saxony and hypothesize frequent exposure to animals based on serological investigations. Hence, its potential risk to human and animal health requires further investigation.

## 1. Introduction

The emergence of new diseases is an increasing threat to human and animal health. Worldwide changes, such as climate change and biodiversity loss, can facilitate the spread of infectious diseases since these factors may favor competent host species [1,2,3]. Moreover, trade and travel also increase the risk of introducing pathogens into new areas [4]. Approximately 60% of known human pathogens are of zoonotic origin, of which a considerable portion is transmitted by arthropod vectors, such as mosquitoes or ticks [5]. Ticks are obligate blood-feeding ectoparasites, which can serve as vectors for various pathogens such as protozoa, bacteria and viruses [5] affecting humans, domestic animals and wildlife [6]. The number of tick-borne diseases is steadily growing and, therefore, becoming an increasing risk for global health [7,8]. Surveillance programs are of crucial importance in order to be able to install appropriate control measures for novel pathogens [4]. Via high-throughput sequencing surveillance programs, various new viral pathogens were discovered in ticks in the past few years [9,10,11].

Among these newly discovered viruses are the Jingmen tick viruses (JMTV), which were first discovered in *Rhipicephalus microplus* ticks collected between 2010 and 2011 in the Jingmen region (province Hubei), China [12]. The Jingmenvirus group consists of enveloped, positive-sense single-stranded (+ss) RNA viruses [12,13]. These viruses have four to five segments, of which two are related to non-structural protein genes of the unsegmented family of *Flaviviridae* (NS5- and NS2b-NS3-like) [12,14]. Jingmenviruses are therefore designated as “flavi-like viruses” [13]. Subsequent reports demonstrated the importance of JMTV as globally distributed viruses with the potential to infect ticks, insects and diverse vertebrate hosts, including humans [12,13,15,16,17,18,19,20,21,22,23,24,25,26].

For instance, anti-JMTV antibodies were found in blood samples from cattle collected in China and Brazil [12,19], and Jingmenviruses RNA was detected in the sera of patients in Kosovo [15]. Additionally, JMTV could be detected in skin biopsies and detached ticks from patients with local and systemic symptoms [27]. Interestingly, a direct association with human disease has been reported for Alongshan virus (ALSV), a JMTV-related virus with 23.7–74.9% homology to JMTV. ALSV was isolated from the blood samples of patients presenting with febrile illness in China [28]. Serological screenings of livestock in the same region demonstrated the presence of anti-ALSV antibodies as well as ALSV RNA in cattle and sheep serum samples [29]. Apart from China, ALSV was also recently described in ticks from Finland, France and Russia [21,22,24]. Analyses of Jingmenvirus genomes furthermore indicate that some of them are arboviruses since tick-borne and mammal-borne strains can be assigned to the same subclades [22]. Phylogenetic analyses of the NS5-like and NS3-like genes revealed that tick and mammalian isolates of Jingmenviruses are more closely related to each other than isolates from insects, indicating that viruses found in mammals are probably tick-borne. Hence, tick-borne Jingmenviruses are prime candidates for a new group of arboviruses [14].

Arboviruses require suitable vector species for viral transmission. Jingmenviruses have been found in multiple tick genera, including *Rhipicephalus*, *Ixodes*, *Haemaphysalis*, *Amblyomma* and *Hyalomma* [12,21,22,23,26,30,31]. Differentiating between possible vector species and non-susceptible species is crucial, as finding a virus in a tick does not prove its vector competency [32]. For example, some species, such as *Dermacentor silvarum*, do not function as suitable vectors [33]. Regarding vector competency, specific aspects must be verified to clearly state the possible viral transmission by the investigated tick species. This includes, for example, the successful replication of JMTV in the salivary glands of *Ixodes persulcatus*, resulting in possible JMTV transmission by tick saliva [27]. In addition to the successful horizontal transmission of arboviruses, the persistence of the virus in the vector species is important for virus maintenance [34]. For Jingmenviruses, it has already been shown that vertical transmission between the tick’s developmental stages might be possible [30,35]. Therefore, investigating other vector-competent species and possible transmission routes for Jingmenviruses is of further interest.

The discovery of Jingmenviruses in Europe prompted us to conduct a survey for Jingmenvirus-related sequences in questing ticks, ticks attached to wild game animals, as well as in the blood and serum samples of both wild game and domestic animals in Lower Saxony, Germany. To further understand the infection cycle of this Lower Saxonian ALSV and its host range, we established a tick infection model and utilized a Luciferase Immunoprecipitation System (LIPS) Assay to analyze the serum samples of potential host species for antibodies. 

## 2. Materials and Methods

### 2.1. Tick Sampling

The ticks were collected in various regions in the German federal state of Lower Saxony from hunted game animals (roe deer, red deer, fallow deer and wild boar) or from the vegetation using the flagging method. Due to the many different hunting areas, the hunted animals were assigned to three major regions (Wolfsburg, Hanover and Harz) according to the forest department system of the Lower Saxony State Forest (*Niedersächsische Landesforsten*, summarized in Table 1).

The ticks were stored for a maximum of 7 days at 4 °C until further handling. In addition, the ticks were sampled by flagging the low vegetation in 2018 at various locations in Lower Saxony as part of a tick-borne encephalitis virus (TBEV) survey with the Lower Saxonian Health authorities (NLGA) [36] (locations in Table S1) as well as in a study for pathogen abundance in ticks at locations in the federal states of Lower Saxony, Bremen and Hesse [37] (locations in Table S2).

For artificial infection studies, adult female *Ixodes ricinus* (*I. ricinus*) ticks were collected by flagging in Ahltener Forest, located in the Misburg, Hanover region. In addition, adult *Dermacentor reticulatus* (*D. reticulatus*) ticks were collected in Wülferode in Hanover, near the Mittelland Canal. The locations of flagging are shown in Table S2. These ticks were stored in a desiccator (Kartell, Novigilo, Italy) at room temperature with a natural day and night cycle and a humidity of about 99% until infection.

### 2.2. Collection of Tick Saliva

The saliva of nearly engorged ticks (*Ixodes* spp.) sampled from hunted game animals (hunting event 2019) was collected as previously described [38]. Briefly, the ticks were placed on a glass slide and fixated with scotch tape. The tick’s hypostome was inserted into a fixated capillary, and 3–5 µL pilocarpine hydrochloride (VWR International, Radnor, PA, USA) was pipetted on the tick’s dorsum. The saliva was collected in a period of 4 h, 0.1 volumes of protease inhibitor cocktail (BIOZOL Diagnostics, Eching, Germany) was added, and the samples were stored at −80 °C until further processing. For PCR testing, the saliva samples from the ticks that had been collected from the same animal were pooled (overview of pools in Table S3).

### 2.3. RNA Isolation

Then, 1 to 60 ticks (per animal) were pooled by collection site and date (overview of all pools in Tables S4 and S5). For homogenizing, three 3 mm steel beads and 500 µL of cell culture medium (Leibowitz´s L-15 or MEM Eagle, Thermo Scientific or ROTI^®^Cell DMEM High Glucose, Carl Roth) were added to each pool. The ticks were disrupted using a TissueLyser II (Qiagen, Hilden, Germany), 20 Hz, 3 min. The homogenates were clarified by centrifugation, and the total RNA was extracted from the homogenates using the QIAamp Viral RNA Mini kit (Qiagen, Hilden, Germany) or the NucleoSpin^®^ RNA virus kit (Macherey-Nagel, Dueren, Germany), according to the manufacturer’s instructions. For RNA extraction from the whole-blood samples, we used a QIAamp Cador Pathogen kit (Qiagen, Hilden, Germany), and for the serum samples and tick saliva, we used a QIAamp Viral RNA Mini kit (Qiagen, Hilden, Germany), following the manufacturer´s instructions.

### 2.4. RT-PCR for Detection of NS5-like Gene and Capsid/Membrane Gene of ALSV and RT-qPCR for Virus Quantification

The whole genome of the ALSV strain Harz Mountain was obtained by using next-generation sequencing (MW094149.1; MW094151.1; MW094148.1; MW094150.1). Based on these sequences of each segment, primers targeting the NS5-like gene were designed for the screening of the samples. The primers used for the detection of ALSV included the forward primer (5′-ATAATCCAGTACCTCCCAGCCG-3′) (nt position 1704) and the reverse primer (5′-CCCCGATGAAACCTGTCCTCTG-3′) (nt position 2039), which target a 334 bp region of the NS5 gene of ALSV. The samples were analyzed using the Qiagen One-Step RT-PCR kit according to the manufacturer’s instructions. Sanger sequencing of positive PCR products was conducted using the forward primer (Eurofins Scientific, Luxemburg). In addition, the positive pools were with primers targeting a 447 bp fragment of the capsid/membrane gene of ALSV using the forward primer (5′-GATGAGGCTAGGGACTTGTTCC-3′) (nt position 1201) and the reverse primer (5′-GTCAGCAGCATCCTAGCCACAT-3′) (nt position 1649) using and the Qiagen One-Step RT-PCR kit, and the positive samples were sequenced, as described above.

For the quantification of viral particles by Sybr RT-qPCR, the Luna^®^ Universal One-Step RT-qPCR Kit (New England Biolabs, Ipswich, MA, USA) was used with the NS5 primer pair. Therefore, a standard curve (10^6^–10^3^ RNA copies) based on the NS5-like gene was prepared.

### 2.5. Virus Cell Culture Experiments

RT-PCR-positive *I. ricinus* homogenates were used for virus cultivation experiments in mammalian (A549, BHK21, VeroE6) and insect (C6/36) cell lines. Therefore, the cells were inoculated with 100 µL of sterile filtrated tick homogenate (diluted 1:10 in minimal essential medium (MEM)) and incubated for one hour at 37 °C (mammalian cells) or 28 °C (insect cells). Afterward, the cells were washed three times with PBS. For cultivation, MEM (mammalian cells) or Schneider’s medium (insect cells) supplemented with 2% fetal bovine serum (FBS) and antibiotics (penicillin/streptomycin, Pan Biotech; gentamicin/amphotericin, Thermo Fisher) was added. The negative controls were inoculated with MEM or Schneider’s medium. The cells were investigated for the presence of a cytopathic effect (CPE) over a period of 14 days, and the samples for virus detection were taken on day 14.

### 2.6. In Vivo Tick ALSV Injection Experiments

For the investigation and establishment of a tick infection model, adult female *I. ricinus* ticks were injected with virus-positive tick homogenate derived from *I. ricinus.* Either 10^3^ or 10^6^ viral copies per tick were injected into the coxae of the 4^th^ leg pair using Nanoject II (Drummond Scientific Company, Broomall, PA, USA). The ticks were incubated at room temperature (approx. 21 °C) in a desiccator (Kartell, Novigilo, Italy) with a humidity of about 99% and a natural day and night cycle until sampling. At each time point (0, 2, 4, 7, 14 and 21 days post-infection), five ticks were examined for viral RNA copy numbers. Infection experiments with *I. ricinus* were repeated three times. In addition, adult female and male *D. reticulatus* ticks were injected with virus-containing tick homogenate derived from *I. ricinus* (10^6^ viral copies per tick). Four ticks (two male and two female) were tested each for viral load at 0, 2, 4, 7, 14 and 49 days post-infection.

### 2.7. In Vitro ALSV Feeding Experiments

The feeding experiments were conducted with an in vitro feeding system, according to Liebig et al. [39], which is based on the system of Kröber & Guerin [40]. Sheep blood in Na-heparin 10 I.U./mL or defibrinated blood was used (Fiebig Nährstofftechnik, Idstein, Germany).

To determine if the virus replicates after ingestion and spreads in *I. ricinus* ticks, the blood was spiked with diluted virus-positive *I. ricinus* homogenates at each blood change throughout the experiment. Visibly engorged female ticks were collected from the membrane after 5 days of feeding and incubated for 1–7 days after the termination of the feeding experiment, depending on survival. After incubation, the peripheral body parts (leg or mouthparts) were separated from the ticks to identify virus dissemination. The samples were homogenized and tested via RT-qPCR, as described above. Overall, five trials were conducted: two in July 2020, two in October 2020 and one in September 2022. In July 2020, 10^6^ ALSV RNA copies/mL, and in October 2020 and September 2022, 10^8^ ALSV RNA copies/mL were added to the blood upon every blood change twice a day.

### 2.8. Virus Transmission Experiments

To verify the transmission of ALSV during the blood meal, female *I. ricinus* ticks were injected with 1.2 × 10^6^ ALSV RNA copies/tick. The ticks were incubated for 7 days prior to blood-feeding experiments. Then, 10 infected female and 10 uninfected male ticks were placed in four feeding chambers each (No. 5–8). Samples were taken with every blood change over a period of 3½ days, after 14 h incubation and after 10 h incubation each day. The RNA was isolated using the QIAamp cador Pathogen Mini Kit following the manufacturer’s protocol (QIAGEN, Hilden, Germany) and tested via RT-qPCR, as described above.

### 2.9. Collection of Animal Serum

The animal serum was collected at hunting events during 2017–2019 in the Lower Saxony State Forest (*Niedersächsische Landesforsten*) from shot game animals. Therefore, VACUETTE tubes, coated with sodium heparin (Greiner Bio-One, Kremsmünster, Austria), were placed in the body cavity of opened hunted game animals and filled with blood. The blood samples were centrifuged at 1000× *g* for 1 min, and the serum was taken and stored at −80 °C until further processing.

Additionally, the samples were obtained in collaboration with the Lower Saxony State Office for Consumer Protection and Food Safety (LAVES, Hanover and Oldenburg) from different areas of Lower Saxony (collected 2017–2020). The samples were generated either by veterinarians of veterinary clinics (University of Veterinary Medicine Hanover), e.g., Clinic for Swine, Small Ruminants, Forensic Medicine and Ambulatory Service (domestic animal) or collected during hunting events (wild animals) (the locations are listed in Tables S6–S11). Additionally, surplus serum samples from domestic animals taken for diagnostic purposes by participating veterinary clinics (University of Veterinary Medicine Hanover) were used. The samples from wild animals were taken from the thoracic or abdominal cavities of shot animals. The serum was centrifuged at 3000× *g* for 10 min, extracted and stored at −20 °C until further examination.

### 2.10. Serological Screening by Luciferase Immunoprecipitation System (LIPS) Assay

For the screening by the LIPS assay, 60 μL aliquots of each of the collected serum samples were stored at −20 °C. Fusion proteins were designed based on the consensus sequence for the viral protein VP2 (putative capsid) of the ALSV strains Harz Mountain (MW094151.1) and cloned into a pcDNA3.1 Zeo vector (containing an IL-6 signal peptide as well as the Nano Luciferase) using the HiFi DNA Assembly Kit (New England Biolabs, Ipswich, MA, USA). Fusion proteins were expressed in transfected Cos-1 cells. Supernatants were collected, and the relative light unit (RLU) was determined.

An LIPS assay was performed with a modified protocol as described [41]. In brief, the fusion proteins were diluted to 500,000–1,000,000 RLU and then mixed in a ratio of 1:1 with PBS. From this fusion protein mixture, 100 μL were pipetted in each well of a 96-well plate. Afterward, 1 µL of undiluted serum per well was added for the assay. For each sample, three replicates were used with specific Luciferase baits and the control luciferase. The plate was incubated on a rotary shaker for 1.5 h. Fifteen minutes before the end of the incubation time, 10 μL of protein A- or G-beads (Fisher Scientific, Hampton, NY, USA) were pipetted in each well of a white 96-well filter plate. After the incubation time, 90 µL of the fusion-protein–serum mixture was transferred to the filter plate (Merck, Darmstadt, Germany) and incubated on a rotary shaker for 1 h. Next, the filter plate was washed on a vacuum device three times with 200 µL of Buffer A (50 mM Tris, 100 mM NaCl, 5 mM MgCl_2_, 1% Triton X-100, pH 7.5) and once with PBS. Then, 50 µL of the Nano-Glo^®^ Luciferase Assay Substrate (1:50 dilution in Nano-Glo^®^ Luciferase Assay Buffer) (Promega, Madison, WI, USA) was added to each well, and the luminescence was measured in the TECAN Infinite^®^ 200 PRO (Tecan Group, Männedorf, Switzerland). For the evaluation of the samples, the mean values and the standard deviation of the triplicates were calculated, and the samples over the mean value +5 standard deviations were classified as positive.

## 3. Results

### 3.1. Prevalence of ALSV in Lower Saxony

From 2017 to 2019, we sampled 984 *Ixodes* spp. ticks from 84 animals (wild boar, roe deer, red deer and fallow deer; detailed information related to species and tick numbers are available in Table 1 and Table S5) and 1766 questing *Ixodes* spp. ticks collected using the flagging method (Locations in Tables S1 and S2). All of these ticks were tested for ALSV RNA using the RT-PCR for the detection of the NS5-like gene. The ticks were pooled as described (Section 2.3, Tables S4 and S5). Out of the 84 tested tick pools from game animals (one pool equals one tested animal), we found 39 pools (46.43%) positive for ALSV NS5-like RNA (Table 2, complete overview Table S5). Further, the PCR fragment generated by the RT-PCR targeting the NS5-like gene was analyzed by Sanger sequencing and sequence analysis confirmed that the viral sequences belong to a Alongshan virus (MW094149.1; MW094151.1; MW094148.1; MW094150.1), which we named Alongshan strain “Harz Mountain”, according to the location of first detection the natural reserve “Harz”. Of the 147 questing tick pools, 10 pools (6.80%) were positive by both RT-PCRs. Positive questing tick pools were found at the site of Rauher Busch (five pools), Barsinghausen (two pools), Nienburg (one pool), Cuxhaven (one pool) and Emen (one pool). Of these 10 positive pools, seven were from nymphs (total 8.14%) and three from adult ticks (total 4.92%). An overview of the results is shown in Table S4.

Furthermore, we tested 536 serum samples as well as the whole blood from wild boar (465 samples), roe deer (29 samples) and red deer (42 samples) using the NS5-like RT-PCR. One red deer serum sample (0.19% overall, 2.38% from red deer samples) from the region of Staufenberg was found to be positive by PCR.

### 3.2. Cell Culture Experiments

All of the cell lines used showed normal growth over a period of 14 days post-infection, and no CPE or morphological abnormalities were observed. Additionally, viral RNA could be detected in two consecutive passages on BHK21, A549 and C6/36 cells, but the RT-qPCR of the cell culture samples displayed no increase in ALSV RNA copy numbers during the course of infection, indicating that viral replication had not occurred in the tested cell lines.

### 3.3. In Vivo Tick Infection Experiments

**Figure 1:**
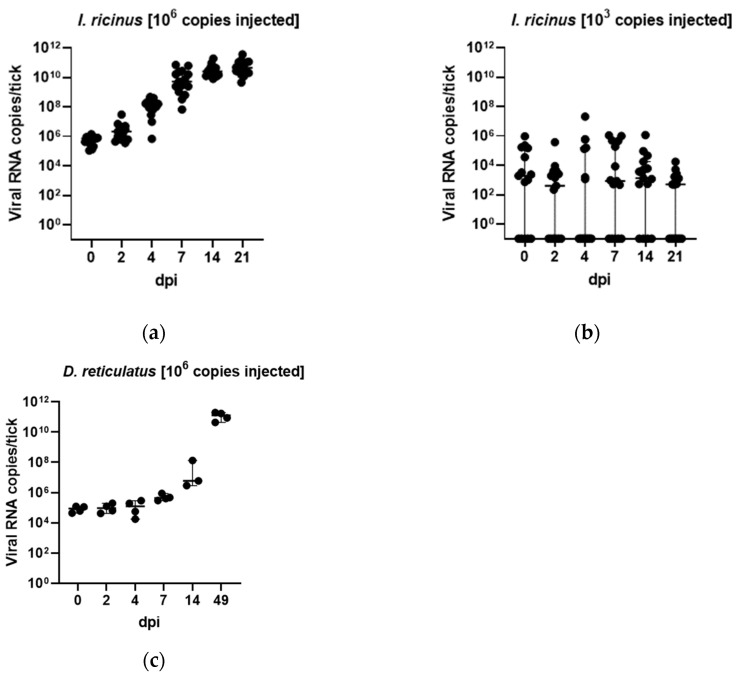
Growth kinetics of the ALSV copy number per tick over a period of 21 or 49 days. (**a**): Growth kinetics of *I. ricinus* with 10^6^ copies inoculum; (**b**): Growth kinetics of *I. ricinus* with 10^3^ copies inoculum. The experiment was repeated three times for *I. ricinus*. (**c**): Growth kinetics of the ALSV copy numbers per *D. reticulatus* tick over a period of 49 days and inoculum of 10^6^ copies/tick. The experiment was conducted once.

Next, we aimed to analyze the ability of ALSV to replicate in the potential vector tick *I. ricinus* and *D. reticulatus*. The ticks injected with ALSV RNA-positive tick homogenates were tested for ALSV RNA copy numbers at days 0, 2, 4, 7, 14 and 21. We found a high increase in viral RNA copies between day 2 and 7 in *I. ricinus* ticks injected with 10^6^ viral copies per tick with values of up to 10^10^ copies/tick, whereas the increase in viral copies from day 7 to 21 was less intense (Figure 1a). In the group injected with 10^3^ viral copies per tick, an overall increase was not observed (Figure 1b), and even many samples were negative for ALSV RNA at the given time points. In *D. reticulatus* ticks, a slight increase in ALSV RNA copies was observed between day 0 and 7 and day 14. The highest viral RNA copy numbers were detected at day 49, with values up to 10^11^ copies/tick (Figure 1c).

### 3.4. In Vitro Feeding Experiments

After we established that ALSV replicates in our artificial infection model, we aimed to analyze the viral replication after oral infection via blood feeding. *I. ricinus* ticks were fed on blood spiked with ALSV positive homogenates in three different experiments. In contrast to experiment 1, in experiments 2 and 3, the input of ALSV RNA copy numbers increased from 10^6^ to 10^8^ ALSV RNA copies per ml of blood. The ticks were collected upon full engorgement or on the day of death. Overall, six body samples out of the eight tested samples were positive for ALSV RNA (Table 3). Furthermore, all of the tested leg samples in the experiment in September 2022 tested positive for ALSV. All of the negative controls were negative for ALSV RNA.

**Figure 2:**
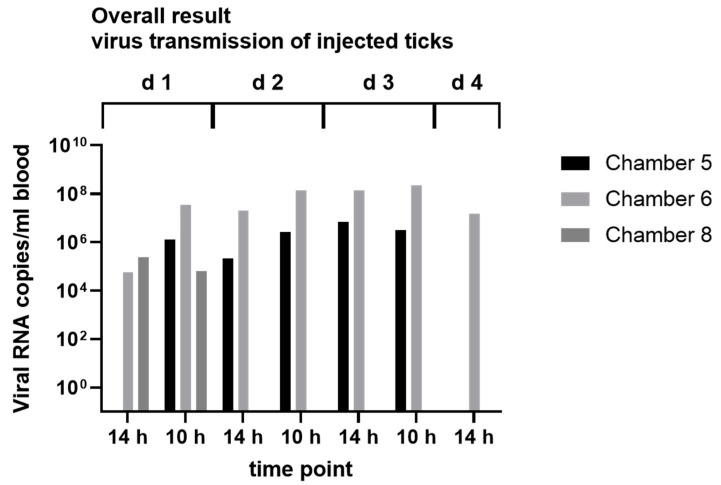
Results of virus transmission experiments with ALSV-injected ticks. ALSV RNA copies/mL blood were measured on 7 different time points (1–7) after 10 h (sampling in the afternoon) or 14 h (sampling in the morning) of incubation of ALSV-injected ticks in feeding chambers 5, 6 and 8. Feeding experiments lasted 3½ days.

Furthermore, we were able to detect ALSV RNA in the blood samples from three of the four used feeding chambers in our virus transmission experiments. Two of these chambers showed positive blood samples over a period of three days (Figure 2), indicating that ALSV was excreted via the tick saliva in the blood meal. To confirm the presence of ALSV in tick saliva, we also analyzed saliva from engorged ticks collected from red deer from the Harz region. We detected ALSV RNA in four out of the seven tested ticks (Table S3).

### 3.5. LIPS Assay

**Figure 3:**
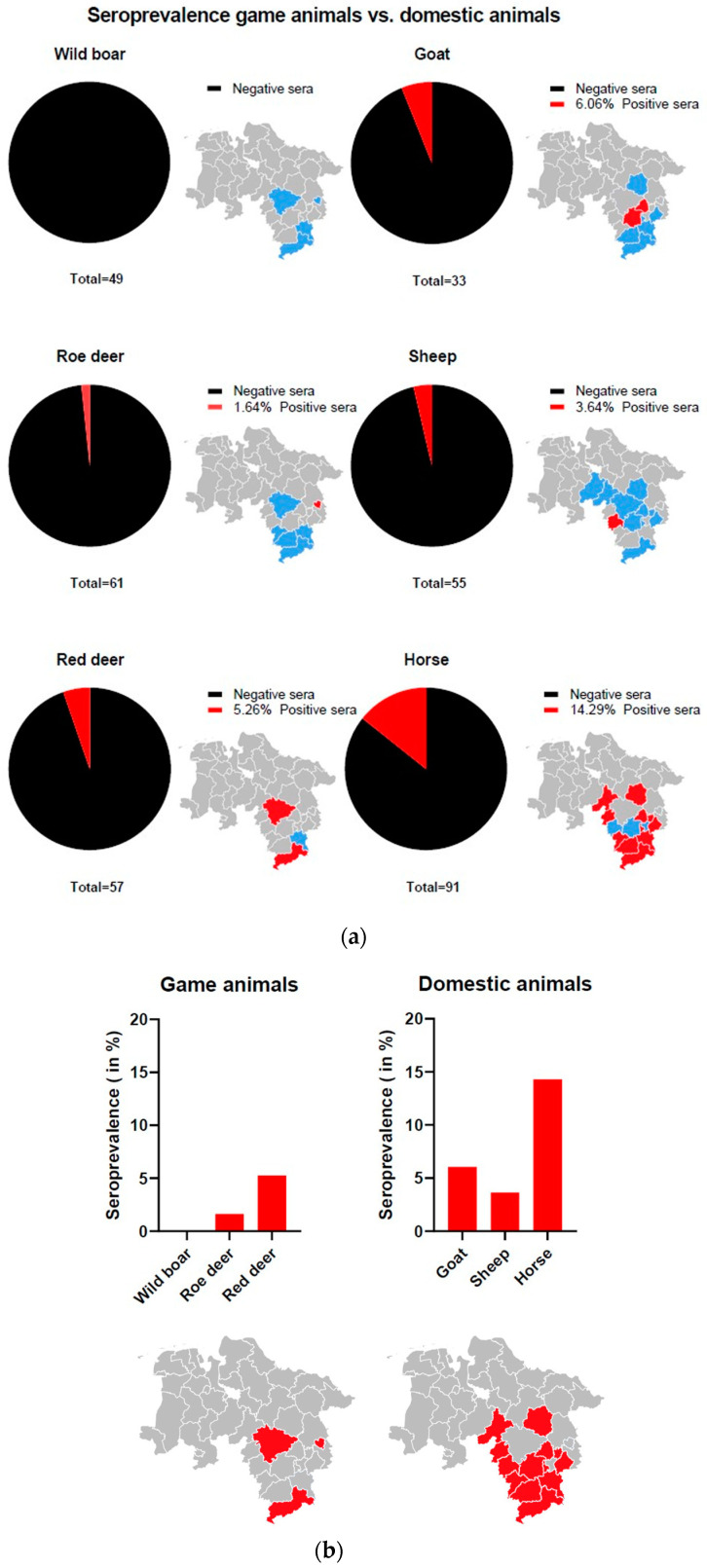
(**a**) Seroprevalence of wild and domestic animals based on LIPS data (red: positive serum sample(s) in this area; blue: serum samples screened in this area; grey: not screened); (**b**) overall seroprevalence of domestic or wild animals. Detailed map information in Figure S1. Maps were created in R v. 4.1.0 with administrative boundaries retrieved from the Global Administrative Areas Database (gadm.org).

**Figure 4:**
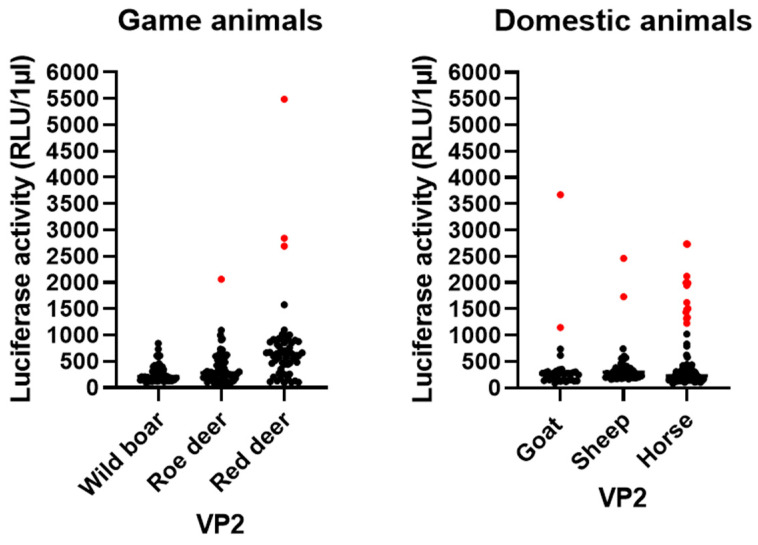
Results of LIPS assay based on 167 animal sera from game animal (wild boar, red deer and roe deer) samples and 179 domestic animal (goat, sheep and horse) samples for the VP2 of ALSV in RLU/µL. Total dataset shown in Tables S4–S9.

To test if ALSV is frequently transmitted to the hosts, we analyzed 167 serum samples from wild animals (49 wild boars, 61 roe deer and 57 red deer) and 179 serum samples from domestic animals (33 goat, 55 sheep and 91 horse) from different regions of Lower Saxony (Tables S4–S9) by LIPS assay for the presence of VP2-specific antibodies. An overview of seroprevalences and RLU/µL is shown in Figure 3 and Figure 4. Three red deer sera (5.26%) showed elevated RLU values and were classified as positive. Of those, two samples were from the Harz and one sample was from the region of Hanover. Furthermore, one roe deer sample (1.64%) from Wolfsburg was classified as positive. No wild boar serum showed significantly elevated RLU and, consequently, all were classified as negative. Regarding the serum samples from domestic animals, two goat serum samples (6.06%) from Hildesheim and Peine, two sheep serum samples (3.63%) from Hameln and 13 horse serum samples (14.29%) from several locations (Northeim, Göttingen, Goslar, Nienburg, Holzminden, Braunschweig, Celle, Schaumburg, Wolfenbüttel and Peine) were classified as positive. Taken together, a broad range of seropositive animals was found, indicating the transmission of ALSV to local game and domestic animals.

## 4. Discussion

Environmental changes due to global warming and biodiversity loss, as well as increased travel, favor the spread of pathogens and increase the risk to public health [1,2,4]. Therefore, surveillance and establishing infection models for new and emerging pathogens are important to gain more knowledge about its maintenance, transmission and spread. Especially Next-Generation Sequencing (NGS) has led to the discovery of many new pathogens, most of which are only known by their sequence, but data concerning host range, vectors and transmission cycles are lacking [42].

One of these new potential pathogens is the group of Jingmenviruses [12], including JMTV and ALSV, which are associated with disease in humans [27,28]. The discovery of JMTV and ALSV in Europe [21,22] prompted us to investigate the potential presence of these viruses in Lower Saxony, Germany. We were able to detect ALSV in immature tick stages and adult male and female ticks, indicating that there might be a transstadial transmission of the virus in tick populations. Furthermore, the finding of JMTV in larvae of *Amblyomma testudinarium* in Japan suggests vertical transmission of the virus [35]; moreover, vertical transmission was shown in *Haemaphysalis longicornis* [30], arguing for the natural maintenance of these viruses in the tick host. However, such a transmission cycle needs to be confirmed in an experimental setting. Regarding the transmission cycle of ALSV, we discovered its viral genomes in the saliva of partially engorged *I. ricinus* ticks removed from wild game animals. This aligns with previous findings of Mogiana tick virus (MGTV) sequences in the cDNA library of *Rhipicephalus microplus* salivary glands [17] and the replication of JMTV in the salivary glands of *Amblyomma javanense* [27]. Additionally, the detection of the ALSV genome in a serum sample from a red deer suggests its transmission to mammalian hosts through blood feeding.

To analyze the transmission cycle of ALSV in more detail and to confirm *I. ricinus* as a vector, we established an infection model for ALSV in *I. ricinus* collected from Hanover. In addition, we performed injections with *D. reticulatus*, which is, after *I. ricinus*, one of the most common tick species in Germany [43]. By using injection into the coxa, viral replication was observed within *I. ricinus* ticks over a period of 21 days in ticks injected with 10^6^ ALSV RNA copies but not with the low dose of 10^3^ ALSV RNA copies per animal. Furthermore, we observed a delayed replication of ALSV between 21 and 49 days post-injection in *D. reticulatus* injected with 10^6^ ALSV RNA copies per animal. The lack of overall replication of the *I. ricinus* group injected with 10^3^ copies could be explained, with too low a number of infectious viral particles in the injected inoculum since we did not quantify infectious viral particles but used the number of viral RNA copies as a proxy for the infective dose. However, the ratio of infectious versus non-infectious virus particles can vary between virus species [44], and 10^3^ RNA copies might refer to a very low number or absence of infectious particles. Additionally, the minimal infective dose (MID) can be different between pathogens and vary from one to many thousands of infectious units [45]. However, the replication of the virus in *I. ricinus,* as well as in *D. reticulatus* ticks, provides the first indications of vector competence and is also a suitable method to generate high amounts of the virus. Since injection does not simulate the natural oral uptake of the virus during feeding [46], artificial feeding experiments were conducted. Positive tick body samples confirmed virus uptake, and at least three virus-positive leg samples confirmed virus spread in the tick. Moreover, the presence of ALSV in the blood from chambers infested with ALSV-infected ticks highlights the transmission via tick saliva. The possible transmission via tick saliva is further enforced by the finding that partially engorged ticks (*Ixodes* spp.) collected from game animals excreted ALSV in an artificial salivation assay.

In addition to virus propagation in vivo, in vitro infection systems, such as a cell-culture-based system, could be interesting in analysing the properties of viral infection and replication. Thus far, we were not able to grow our ALSV isolates in the cultured cells of insect or mammalian origin. During the incubation period, the cells showed normal growth and did not indicate any CPE or other abnormality due to viral infection. We observed viral RNA in two consecutive passages on BHK21, A549 and C6/36 cells but found no increase in viral RNA copy numbers by qPCR and cannot exclude that this is due to the carryover of PCR-positive tick homogenates used for inoculation, as also discussed by Kobayashi et al. (2021) [35]. This corresponds with the cell culture findings of Qin et al. (2014) [12], which inoculated C6/36 and DH82 cells (canine macrophage cell line) with JMTV, Kuivanen et al. (2019) infecting Vero, SK-N-SH and CRL-2088 cells [21] and Kobayashi et al. (2021) [35]. Successful replication of ALSV was already shown in tick cell lines [24], which could be undertaken in further experiments.

Interestingly, we also found remarkable differences in ALSV detection between ticks collected by the flagging method, which are usually questing unfed ticks and partially engorged ticks sampled from shot game animals. The ALSV infection rates in partially engorged ticks were 6.7-fold higher than in unfed ticks. This might be due to enhanced viral replication in the tick after a blood meal, as been described for TBEV [47]. Alternatively, a transmission of the virus via co-feeding of infected and uninfected ticks on the same animal host could be the reason for the high ALSV detection rates in ticks collected from animals, as described for TBEV [48] and also suggested by Qin et al. (2014) for JMTV [12].

ALSV and JMTV have been associated with disease in humans [27,28]. The fact that JMTV-positive ticks were found in symptomatic patients reinforces the suspicion that this virus is transmitted by ticks [27]. Additionally, spillover from game animals to humans is also possible when they come into contact [49]. To date, we detected ALSV RNA in one serum sample of a red deer, providing evidence for viral transmission to mammalian hosts. We did not detect viral RNA in any other sample; however, some of the tested samples were derived from other studies, and thus, storage might not be optimal for viral RNA detection. Therefore, the presence of viral RNA in wild animals might be even higher than shown in this study. Furthermore, screening for antibodies against ALSV in the blood samples of wild animals and livestock in Lower Saxony indicated contact as well as the replication of the virus in several animal species. Antibodies were detected in red deer, roe deer, goat, sheep and horse, showing a broad host range of ALSV. Similar studies have shown a wide distribution of anti-JMTV antibodies in cattle in China and Brazil [12,19]. Sheep samples could also be tested positive for other JMVs beforehand [22,29]. The plasma examination of a non-human primate (red colobus monkey, *Procolobus rufomitratus*) from Uganda as well as blood samples of Kosovan patients infected with Crimean–Congo hemorrhagic fever virus (CCHFV), revealed the presence of Jingmenvirus genome [15,16]. In addition, anti-ALSV antibodies, as well as ALSV RNA, were detected in livestock in China during the first grazing season [29].

Based on these reports, including the here presented study, JMT viruses could be proposed as new emerging tick-borne viruses.

## 5. Conclusions

We found ALSV in ticks, tick saliva and a serum sample of red deer in Lower Saxony, Germany. We could cultivate this virus in vivo and prove replication in ticks. Virus transmission, uptake and spread were demonstrated, suggesting vector competence of *I. ricinus* and *D. reticulatus*. Furthermore, we could show a broad host range, including domestic and wild animals. Our results provide initial insights into the natural transmission cycle and maintenance of the virus.

## Figures and Tables

**Table 1:** Overview of sampled game animals and ticks per region.

Region	Sampled Animals	Number of Ticks
Wolfsburg	25	254
Hanover	11	40
Harz	48	690
Total	84	984

**Table 2:** Results of RT-qPCR tests for ALSV from wild game tick pools separated by sampling location and animal species (shown in positive pools/total pools).

Region	Pools	Positive Pools [Positive/Total]	Red Deer [Positive/Total]	Roe Deer [Positive/Total]	Fallow Deer [Positive/Total]
Wolfsburg	25	8/25	0/2	8/22	0/1
Harz	43	27/43	27/41	0/2	0
Hanover	16	4/16	3/12	1/4	0
Total	84	39/84	30/55	9/28	0/1

**Table 3:** Results of feeding experiments of *I. ricinus* ticks with ALSV infected blood.

Date	Virus Copies/mL Blood	Incubation [Day]of Different Tick Samples	Positive Body [Positive/Total]	Positive Peripheral Body Part [Positive/Total]
July 2020	10^6^	1, 7	1/2	0/2
October 2020	10^8^	2, 7, 7	2/3	0/3
September 2022	10^8^	5, 5, 5	3/3	3/3
Total			6/8	3/8

## Data Availability

Not applicable.

## Supplementary Materials

The following supporting information can be downloaded at: https://www.mdpi.com/article/10.3390/microorganisms11030543/s1, Tables S1–S2: Locations of flagging; Table S3: Overview of tick saliva pools sampled from ticks found on wild animals and NS5 PCR results of serum samples from wild animals and tick samples; Table S4: Overview of flagged ticks from several locations with information regarding life stages and/or sex and amount of ticks per pool; Table S5: Overview of pools by location and by the sampled game animal with NS5 PCR results and number of ticks per pool; Table S6–S11: Overview of tested serum samples for ALSV VP2 by LIPS assay; Figure S1: Card of Lower Saxony with Regions.
