# Supplementary material for: Detection and Characterization of Alongshan Virus in Ticks and Tick Saliva from Lower Saxony, Germany with Serological Evidence for Viral Transmission to Game and Domestic Animals
Source: Microorganisms. 2023 Feb 21;11(3):543. doi: 10.3390/microorganisms11030543 (PMC10055853; doi:10.3390/microorganisms11030543)
Supplement: Supplementary file 1 [file microorganisms-11-00543-s001.zip › microorganisms-2177228-supplementary.pdf]

Supplements

Table S1: Locations of flagging. Ticks were flagged in frame of the Boelke et al. (2019) study.

Region	Coordinates
Rauher Busch	N52◦340; E8◦480
Barsinghausen Mooshütte	N52◦190; E9◦230
Cuxhaven	N53°43; E9°02
Celle	N52°34; E10°00
Lingen	N52°30; E7°20
Nienburg	N52°38; E9°10

Table S2: Locations of flagging. Ticks were flagged in frame of the Knoll et al. (2021) study.

Region	Area	Coordinates
Hanover	Kolshorn	N52°23; E9°55
	Bockmerholz	N52°18; E9°51
	Ricklingen	N52°19; E9°43
	Misburg	N52°40; E9°84
	Wülferode	N52°34; E9°87
Kassel	Wilhelmshöhe	N51°19; E9°24
	Erlenloch	N51°20; E9°24
	Niederelsungen	N51°23; E9°13
Uelzen	Hamerstorf	N52°55; E10°27
	Ebstorfer Str	N52°58; E10°3
	B4	N52°52; E10°30
Bremen	Oyten	N53°03; E8°59
	Bürgerpark	N53°06; E8°50
	Syke	N52°55; E8°49
Emsland	Lathen	N52°52; E7°19
	Emen	N52°48; E7°16
	Wesuwe	N52°45; E7°11

Table S3: Overview of tick saliva pools sampled from ticks found on wild animals and NS5 PCR results of serum samples from wild animals and tick samples.

Nr.	Sample-ID	Species	Blood sample available	Ticks	NS5 PCR Serum	NS5 PCR Ticks	Location	Amount of saliva
1	5749	red deer	yes	4 (2 female, 2 male)	negative	positive	Kamschlacken	no saliva
2	6044	red deer	yes	3 (2 female, 1 male)	negative	negative	Kamschlacken	no saliva
3	6008	red deer	yes	5 (3 female, 2 male)	negative	positive	Kamschlacken	1 µl
4	6009	red deer	yes	2 (1 female, 1 male)	negative	positive	Kamschlacken	1 µl
5	6014	red deer	yes	3 male	negative	positive	Kamschlacken	no saliva
6	6011	red deer	yes	8 (5 female, 3 male)	negative	positive	Kamschlacken	5 µl
7	6010	red deer	yes	0			Kamschlacken	no saliva
8	6024	red deer	yes	4 (3 female, 1 male)	negative	negative	Kamschlacken	no saliva
9	5117	red deer	yes	4 (2 female, 2 male)	negative	negative	Riefensbeek	1 µl
10	6040	red deer	yes	0			Kamschlacken	no saliva
11	6041	red deer	yes	8 (4 female, 4 male)	negative	negative	Kamschlacken	no saliva
12	4625	red deer	yes	9 (4 female, 5 male)	negative	negative	Riefensbeek	3 µl
13	5118	red deer	yes	4 (2 female, 2 male)	negative	negative	Riefensbeek	6 µl
14	6015	red deer	yes	0			Kamschlacken	no saliva
15	*0070	red deer	no	1 (male)		positive	Nationalpark Harz ?	no saliva
16	6007	red deer	no	5 (4 female, 1 male)		positive	Kamschlacken	2.2 µl
				total: 60 ticks				

Table S4: Overview of flagged ticks from several locations with information regarding life stages and/or sex and amount of ticks per pool.

Pool-ID.	Location	Stage or sex	Ticks/pool	NS5 PCR
1	Garten Nienburg	male	1	negative
2	zur Rolle	adult	3	positive
3	zur Rolle	nymph	9	negative
4	Rauher Busch	nymph	20	negative
5	Rauher Busch	nymph	20	negative
6	Rauher Busch	nymph	20	negative
7	Rauher Busch	nymph	20	positive
8	Rauher Busch	nymph	20	positive
9	Rauher Busch	adult	10	negative
10	Rauher Busch	adult	10	positive
11	Rauher Busch	nymph	20	negative
12	Rauher Busch	nymph	20	negative
13	Rauher Busch	adult	10	negative
14	Rauher Busch	nymph	20	positive
15	Rauher Busch	nymph	20	negative
16	Rauher Busch	adult	6	negative
17	Rauher Busch	nymph	20	negative
18	Rauher Busch	nymph	20	negative
19	Rauher Busch	adult	10	negative
20	Rauher Busch	nymph	20	positive
25	Wathlingen	nymph	20	negative
26	Wathlingen	nymph	20	negative
27	Wathlingen	adult	10	negative
28	Wathlingen	nymph	20	negative
29	Wathlingen	nymph	20	negative
30	Wathlingen	nymph	20	negative
31	Wathlingen	adult	10	negative
32	Wathlingen	nymph	20	negative
33	Wathlingen	nymph	20	negative
34	Wathlingen	adult	10	negative
35	Wathlingen	adult	10	negative
36	Wathlingen	nymph	20	negative
37	Wathlingen	nymph	20	negative
38	Wathlingen	nymph	20	negative
39	Wathlingen	nymph	20	negative
40	Wathlingen	nymph	20	negative
41	Wathlingen	nymph	20	negative
42	Wathlingen	adult	6	negative
43	Wathlingen	nymph	20	negative
44	Wathlingen	nymph	15	negative
45	Mooshütte Wald	adult	10	negative
46	Mooshütte Wald	nymph	20	negative
47	Mooshütte Wald	adult	4	negative

48	Mooshütte Wald	nymph	17	negative
49	Mooshütte Feld	adult	10	negative
50	Mooshütte Feld	nymph	20	negative
51	Mooshütte Feld	adult	10	negative
52	Mooshütte Feld	nymph	20	positive
53	Mooshütte Feld	nymph	20	negative
54	Mooshütte Feld	nymph	20	negative
55	Mooshütte Feld	adult, female	3	negative
56	Mooshütte Feld	nymph	20	negative
57	Kammweg	adult	5	negative
58	Kammweg	nymph	20	positive
60	Deister Alm	adult	10	negative
61	Deister Alm	adult	3	negative
64	Deister	adult	9	negative
65	Deister	nymph	20	negative
85	Cuxhaven	nymph	20	negative
86	Cuxhaven	adult	9	positive
87	Cuxhaven	nymph	20	negative
88	Cuxhaven	nymph	20	negative
89	Cuxhaven	nymph	8	negative
90	Cuxhaven	nymph	20	negative
91	Cuxhaven	nymph	20	negative
92	Cuxhaven	adult	1	negative
93	Cuxhaven	nymph	20	negative
94	Cuxhaven	nymph	13	negative
95	Cuxhaven	nymph	20	negative
98	Cuxhaven	nymph	20	negative
103	Cuxhaven	nymph	20	negative
104	Cuxhaven	nymph	20	negative
105	Cuxhaven	adult	4	negative
106	Cuxhaven	nymph	20	negative
107	Cuxhaven	nymph	20	negative
108	Cuxhaven	nymph	20	negative
110	Lingen	adult	3	negative
113	Lingen	nymph	20	negative
114	Lingen	adult	3	negative
115	Lingen	nymph	20	negative
116	Lingen	nymph	20	negative
117	Lingen	adult	3	negative
120	Lingen	nymph	10	negative
121	Lingen	nymph	1	negative
122	Lingen	adult	5	negative
123	Lingen	nymph	20	negative
124	Lingen	nymph	14	negative
125	Lingen	adult	3	negative
126	Lingen	nymph	22	negative

16SK18	Wesuwe	female	2	negative
37SK18	Lathen	nymph	10	negative
56SK18	Emen	nymph	9	positive
72SK18	Ebstorfer Str.	nymph	7	negative
88SK18	Hamerstorf	female	3	negative
108SK18	B4	nymph	14	negative
126SK18	Kolshorn	nymph	6	negative
142SK18	Bockmerholz	female	5	negative
160SK18	Ricklingen	female	5	negative
178SK18	Bürgerpark	female	8	negative
200SK18	Syke	nymph	11	negative
217SK18	Oyten	male	10	negative
236SK18	Erlenloch	nymph	8	negative
252SK18	Niederelsungen	female	10	negative
272SK18	Wilhelmshöhe	male	7	negative
291SK18	Wesuwe	nymph	5	negative
310SK18	Lathen	nymph	15	negative
329SK18	Emen	nymph	18	negative
346SK18	Ebstorfer Str.	male	6	negative
366SK18	Hamerstorf	nymph	9	negative
382SK18	B4	female	4	negative
401SK18	Kolshorn	female	7	negative
420SK18	Bockmerholz	female	4	negative
438SK18	Ricklingen	female	3	negative
456SK18	Bürgerpark	female	3	negative
475SK18	Syke	male	5	negative
495SK18	Oyten	nymph	15	negative
511SK18	Erlenloch	female	4	negative
531SK18	Niederelsungen	nymph	10	negative
549SK18	Wilhelmshöhe	male	5	negative
54SK18	Emen	female	1	negative
55SK18	Emen	male	2	negative
326SK18	Emen	female	2	negative
327SK18	Emen	male	7	negative
328SK18	Emen	nymph	4	negative
604SK18	Emen	female	5	negative
605SK18	Emen	male	9	negative
606SK18	Emen	nymph	10	negative
607SK18	Emen	nymph	14	negative
878SK18	Emen	female	9	negative
879SK18	Emen	male	6	negative
880SK18	Emen	nymph	10	negative
881SK18	Emen	nymph	41	negative
1151SK18	Emen	female	1	negative
1152SK18	Emen	male	1	negative
1153SK18	Emen	nymph	6	negative
1154SK18	Emen	nymph	29	negative

1428SK18	Emen	female	4	negative
1429SK18	Emen	male	4	negative
1430SK18	Emen	nymph	9	negative
1431SK18	Emen	nymph	7	negative
1705SK18	Emen	female	3	negative
1706SK18	Emen	male	5	negative
1707SK18	Emen	nymph	9	negative
1708SK18	Emen	nymph	14	negative
1_2017	Seelhorster Wald	adult	2	negative
2_2017	Seelhorster Wald	adult	2	negative
3_2017	Sulinger Bruch	adult	2	negative
total	147 pools		1766	10 positive

Table S5: Overview of pools by location and by sampled game animal with NS5 PCR results and number of ticks per pool.

Nr.	Date	Sample-ID	Tick species	Location	Ticks/pool*	Host animal	NS5 PCR
1	2017	4140	*Ixodes* spp.	Riefensbeek	10	red deer	positive
2	2017	4140	*Ixodes* spp.	Riefensbeek	10	red deer	positive
3	2017	4141	*Ixodes* spp.	Riefensbeek	11	red deer	positive
4	2017	4142	*Ixodes* spp.	Riefensbeek	7	red deer	negative
5	2017	4143	*Ixodes* spp.	Riefensbeek	7	red deer	positive
6	2017	4713	*Ixodes* spp.	Riefensbeek	25	red deer	positive
7	2017	6781	*Ixodes* spp.	Barsinghausen	1	red deer	negative
8	2017	6787	*Ixodes* spp.	Barsinghausen	3	red deer	positive
9	2017	6672	*Ixodes* spp.	Barsinghausen	3	red deer	negative
10	2017	6695	*Ixodes* spp.	Barsinghausen	1	red deer	negative
11	2017	6056	*Ixodes* spp.	Barsinghausen	2	roe deer	negative
12	2017	6053	*Ixodes* spp.	Barsinghausen	2	red deer	negative
13	2017	6970	*Ixodes* spp.	Barsinghausen	5	red deer	negative
14	2018	010518R	*Ixodes* spp.	Wolfsburg	22	roe deer	positive
15	2018	010518P	*Ixodes* spp.	Wolfsburg	21	roe deer	negative
16	2018	010518D.1	*Ixodes* spp.	Wolfsburg	24	roe deer	positive
17	2018	16709636	*Ixodes* spp.	Waldhof	24	roe deer	negative
18	2018	010718R	*Ixodes* spp.	Wolfsburg	8	roe deer	positive
19	2018	16708415	*Ixodes* spp.	Waldhof	3	roe deer	negative
20	2018	280718J	*Ixodes* spp.	Wolfsburg	9	roe deer	positive
21	2018	210718J	*Ixodes* spp.	Wolfsburg	4	roe deer	negative
22	2018	010718J	*Ixodes* spp.	Wolfsburg	9	roe deer	positive
23	2018	16709648	*Ixodes* spp.	Barnbruch	3	roe deer	positive
24	2018	16708429	*Ixodes* spp.	Rothehof	11	roe deer	negative
25	2018	16708421	*Ixodes* spp.	Hehlinger Holz	29	roe deer	negative
26	2018	16708420	*Ixodes* spp.	Wolfsburg	5	roe deer	negative
27	2018	16708418	*Ixodes* spp.	Waldhof	6	roe deer	negative
28	2018	1670 8417	*Ixodes* spp.	Hehlinger Holz	6	roe deer	negative

29	2018	1670 9627	*Ixodes* spp.	Giebel	6	roe deer	negative
30	2018	1670 9634	*Ixodes* spp.	Danndorf	7	roe deer	negative
31	2018	1670 9641	*Ixodes* spp.	Hohenstedter	7	roe deer	negative
32	2018	1670 9684	*Ixodes* spp.	Barnbruch	8	roe deer	positive
33	2018	1670 9689	*Ixodes* spp.	Giebel	8	roe deer	negative
34	2018	1670 9690	*Ixodes* spp.	Waldhof	8	roe deer	positive
35	2018	0828 (828)	*Ixodes* spp.	Braunlage	60	red deer	positive
36	2018	4485	*Ixodes* spp.	Riefensbeek	28	red deer	negative
37	2018	25907869	*Ixodes* spp.	Fuhrberg	23	red deer	positive
38	2018	25907870	*Ixodes* spp.	Fuhrberg	9	red deer	negative
39	2018	25907876	*Ixodes* spp.	Fuhrberg	23	red deer	positive
40	2018	25907878	*Ixodes* spp.	Fuhrberg	23	red deer	positive
41	2018	25907880	*Ixodes* spp.	Fuhrberg	3	roe deer	negative
42	2018	4084	*Ixodes* spp.	Braunlage	29	red deer	positive
43	2018	4089	*Ixodes* spp.	Braunlage	48	red deer	positive
44	2018	4091	*Ixodes* spp.	Braunlage	26	red deer	negative
45	2018	4095	*Ixodes* spp.	Braunlage	44	red deer	positive
46	2018	0574 (574)	*Ixodes* spp.	Braunlage	33	red deer	positive
47	2018	1935	*Ixodes* spp.	Riefensbeek	2	red deer	positive
48	2018	2261	*Ixodes* spp.	Riefensbeek	1	red deer	negative
49	2018	2262	*Ixodes* spp.	Riefensbeek	29	red deer	positive
50	2018	2386	*Ixodes* spp.	Hohegeiß	10	red deer	positive
51	2018	2387	*Ixodes* spp.	Hohegeiß	10	red deer	negative
52	2018	2389	*Ixodes* spp.	Hohegeiß	10	red deer	positive
53	2018	2391	*Ixodes* spp.	Hohegeiß	10	red deer	negative
54	2018	2388	*Ixodes* spp.	Hohegeiß	10	red deer	negative
55	2018	3324	*Ixodes* spp.	Riefensbeek	2	red deer	positive
56	2018	4484	*Ixodes* spp.	Riefensbeek	15	red deer	positive
57	2018	5022	*Ixodes* spp.	Riefensbeek	10	red deer	positive
58	2018	5024	*Ixodes* spp.	Riefensbeek	20	red deer	positive
59	2018	5025	*Ixodes* spp.	Riefensbeek	11	red deer	negative
60	2018	5027	*Ixodes* spp.	Riefensbeek	9	red deer	positive
61	2018	5028	*Ixodes* spp.	Riefensbeek	3	roe deer	negative
62	2018	0828	*Ixodes* spp.	Braunlage	60	red deer	positive
63	2018	040818J	*Ixodes* spp.	Wolfsburg	6	roe deer	negative
64	2019	5749	*Ixodes* spp.	Kamschlacken	4	red deer	positive
65	2019	6044	*Ixodes* spp.	Kamschlacken	3	red deer	negative
66	2019	6008	*Ixodes* spp.	Kamschlacken	5	red deer	positive
67	2019	6009	*Ixodes* spp.	Kamschlacken	2	red deer	positive
68	2019	6014	*Ixodes* spp.	Kamschlacken	3	red deer	positive
69	2019	6011	*Ixodes* spp.	Kamschlacken	8	red deer	positive
70	2019	6024	*Ixodes* spp.	Kamschlacken	4	red deer	negative
71	2019	5117	*Ixodes* spp.	Riefensbeek	4	red deer	negative
72	2019	6041	*Ixodes* spp.	Kamschlacken	8	red deer	negative
73	2019	4625	*Ixodes* spp.	Riefensbeek	9	red deer	negative
74	2019	5118	*Ixodes* spp.	Riefensbeek	4	red deer	negative

75	2019	*0070	*Ixodes* spp.	Nationalpark Harz	1	red deer	positive
76	2019	6007	*Ixodes* spp.	Kamschlacken	5	red deer	positive
77	2019	92,1	*Ixodes* spp.	Alfeld (Han.)	3	roe deer	negative
78	2019	94,1	*Ixodes* spp.	St. Andreasberg, Harz	5	roe deer	negative
79	2019	01Okt19	*Ixodes* spp.	Söllingen (Wob)	10	roe deer	negative
80	2019	02Okt19	*Ixodes* spp.	Hösseringen (Han.)	10	red deer	negative
81	2019	04Okt19	*Ixodes* spp.	Hösseringen (Han.)	5	red deer	negative
82	2019	05Okt19	*Ixodes* spp.	Calberlah (Wob)	5	roe deer	negative
83	2019	06Okt19	*Ixodes* spp.	Steinhorst (Han.)	5	red deer	negative
84	2019	01Nov19	*Ixodes* spp.	Söllingen (Wob)	5	red deer	negative
84	total				984		39 positive<br>45 negative

Table S6: Overview of tested wild boar serum samples for ALSV VP2 by LIPS assay.
Red: classified positive, green: classified negative, n=49, positive: 0, seroprevalence: 0%.

Sample-ID	Species	Location	Date	VP2-Capsid
0278	wild boar	Beerbusch	2017	284
0445	wild boar	Beerbusch	2017	307
3336	wild boar	Beerbusch	2017	287
3338	wild boar	Beerbusch	2017	456
3372	wild boar	Beerbusch	2017	414
3373	wild boar	Beerbusch	2017	305
3378	wild boar	Beerbusch	2017	397
3381	wild boar	Beerbusch	2017	420
3383	wild boar	Beerbusch	2017	184
3431	wild boar	Beerbusch	2017	214
3448	wild boar	Beerbusch	2017	159
3452	wild boar	Beerbusch	2017	124
3457	wild boar	Beerbusch	2017	188
3460	wild boar	Beerbusch	2017	181
3496	wild boar	Beerbusch	2017	120
4329	wild boar	Riefensbeek	2017	734
4642	wild boar	Riefensbeek	2017	606
4871	wild boar	Wolfsburg	2017	313
4875	wild boar	Wolfsburg	2017	377
4886	wild boar	Wolfsburg	2017	609
4887	wild boar	Wolfsburg	2017	589
4888	wild boar	Wolfsburg	2017	463
4889	wild boar	Wolfsburg	2017	399

6044	wild boar	Barsinghausen	2017	**844**
WS Rundshorn	wild boar	Rundshorn	2017	**98**
WS Wob 24.06.18	wild boar	Wolfsburg	June 18	**207**
WS Wob 24.06.18	wild boar	Wolfsburg	June 18	**183**
WS Wob 24.06.18	wild boar	Wolfsburg	June 18	**195**
WS Wob 24.06.18	wild boar	Wolfsburg	June 18	**341**
L89	wild boar	Osterode am Harz	2019	**184**
L90	wild boar	Osterode am Harz	2019	**208**
L319	wild boar	Osterode am Harz	2019	**228**
L372	wild boar	Springe	2019	**116**
L402	wild boar	Osterode am Harz	2019	**143**
L403	wild boar	Osterode am Harz	2019	**208**
L446	wild boar	Bad Lauterberg im Harz	2019	**157**
L448	wild boar	Osterode am Harz	2019	**163**
L504	wild boar	Osterode am Harz	2019	**295**
L519	wild boar	Osterode am Harz	2019	**243**
L539	wild boar	Osterode am Harz	2019	**150**
L544	wild boar	Osterode am Harz	2019	**215**
L560	wild boar	Bad Grund (Harz)	2019	**129**
L607	wild boar	Osterode am Harz	2019	**119**
L608	wild boar	Osterode am Harz	2019	**112**
L609	wild boar	Osterode am Harz	2019	**187**
L627	wild boar	Bad Harzburg	2019	**340**
L659	wild boar	Osterode am Harz	2019	**150**
L812	wild boar	Osterode am Harz	2019	**125**
Wildschwein Wob 21.12.19	wild boar	Wolfsburg	December 19	**423**

Table S7: Overview of tested roe deer serum samples for ALSV VP2 by LIPS assay.
Red: classified positive, green: classified negative, n=61, positive: 1, seroprevalence: 1.64%.

Sample-ID	Species	Location	Date	VP2-Capsid
3451	Roe deer	Beerbusch	2017	484
4358	roe deer	Riefensbeek	2017	734
6045	roe deer	Barsinghausen	2017	717
6054	roe deer	Barsinghausen	2017	581
6757	roe deer	Barsinghausen	2017	926
6792	roe deer	Barsinghausen	2017	599
Reh Rundshorn	roe deer	Rundshorn	2017	203
Reh Wob	roe deer	Wolfsburg	2017	275
Reh weibl. Wob	roe deer	Wolfsburg	2017	1093
Rehbock Wob	roe deer	Wolfsburg	2017	2061
Bock Wob I	roe deer	Wolfsburg	2018	526
Bock Wob	roe deer	Wolfsburg	2018	997
Jährling 1 Wob	roe deer	Wolfsburg	2018	564
Jährling 13 Wob	roe deer	Wolfsburg	2018	421

Jährling 15 Wob	roe deer	Wolfsburg	2018	425
Jährling A Wob	roe deer	Wolfsburg	2018	303
Bock Wob 23.06.	roe deer	Wolfsburg	June 18	620
Jährling Wob 28.06.18	roe deer	Wolfsburg	June 18	445
Jährling Wob 23.06.18	roe deer	Wolfsburg	June 18	282
Bock Wob 23.06.	roe deer	Wolfsburg	June 18	300
Bock Wob 23.06.18	roe deer	Wolfsburg	June 18	187
Bock Wob 23.06.18	roe deer	Wolfsburg	June 18	308
1	roe deer	Wunstorf	2019	297
2	roe deer	Wunstorf	2019	595
3	roe deer	Wunstorf	2019	905
4	roe deer	Ebergötzen	2019	312
5	roe deer	Ebergötzen	2019	191
6	roe deer	Ebergötzen	2019	354
9	roe deer	Hattorf am Harz	2019	134
10	roe deer	Hattorf am Harz	2019	177
11	roe deer	Hattorf am Harz	2019	263
12	roe deer	Hattorf am Harz	2019	130
13	roe deer	Hattorf am Harz	2019	365
14	roe deer	Hattorf am Harz	2019	152
15	roe deer	Hattorf am Harz	2019	229
16	roe deer	Hattorf am Harz	2019	141
17	roe deer	Hattorf am Harz	2019	158
18	roe deer	Hattorf am Harz	2019	270
L1055	roe deer	Gleichen	2019	636
L1063	roe deer	Katlenburg-Lindau	2019	520
L1064	roe deer	Katlenburg-Lindau	2019	628
Rehbock Wob 21.12.19	roe deer	Wolfsburg	December 2019	278
35	roe deer	Holzminden	May 20	90
37	roe deer	Holzminden	May 20	107
38	roe deer	Holzminden	May 20	82
39	roe deer	Holzminden	May 20	94
40	roe deer	Holzminden	May 20	74
128	roe deer	Göttingen	Autumn 20	91
129	roe deer	Göttingen	Autumn 20	100
134	roe deer	Holzminden	Autumn 20	100
135	roe deer	Holzminden	Autumn 20	114
136	roe deer	Holzminden	Autumn 20	249
137	roe deer	Holzminden	Autumn 20	242
138	roe deer	Holzminden	Autumn 20	477
139	roe deer	Holzminden	Autumn 20	257
172	roe deer	Holzminden	Autumn 20	245
173	roe deer	Holzminden	Autumn 20	228
174	roe deer	Holzminden	Autumn 20	193
175	roe deer	Holzminden	Autumn 20	361
176	roe deer	Holzminden	Autumn 20	291
177	roe deer	Holzminden	Autumn 20	219

Table S8: Overview of tested red deer serum samples for ALSV VP2 by LIPS assay.
Red: classified positive, green: classified negative, n=57, positive: 3, seroprevalence: 5.26%.

Sample-ID	Species	Location	Date	VP2-Capsid
4142	red deer	Riefensbeek	2017	756
4143	red deer	Riefensbeek	2017	876
4633	red deer	Riefensbeek	2017	931
6052	red deer	Barsinghausen	2017	597
6053	red deer	Barsinghausen	2017	601
6056	red deer	Barsinghausen	2017	559
6075	red deer	Barsinghausen	2017	655
6091	red deer	Barsinghausen	2017	784
6695	red deer	Barsinghausen	2017	2842
6758	red deer	Barsinghausen	2017	854
6970	red deer	Barsinghausen	2017	664
Rotwild Rundshorn	red deer	Rundshorn	2017	1575
St. Andreasberg RW Kalb	red deer	St. Andreasberg	2017	901
L1060	red deer	Oberode	2019	651
L1061	red deer	Oberode	2019	518
L1062	red deer	Oberode	2019	444
L1065	red deer	Braunlage	2019	939
L1066	red deer	Braunlage	2019	473
L1067	red deer	Braunlage	2019	484
L1068	red deer	Braunlage	2019	672
L1069	red deer	Braunlage	2019	573
L1070	red deer	Goslar	2019	468
L1071	red deer	Goslar	2019	353
L1072	red deer	Goslar	2019	444
L1073	red deer	Goslar	2019	866
L1074	red deer	Goslar	2019	463
L1075	red deer	Staufenberg	2019	660
L1076	red deer	Staufenberg	2019	674
L1077	red deer	Staufenberg	2019	700
L1078	red deer	Staufenberg	2019	650
5749	red deer	Kamschlacken	2019	919
6044	red deer	Kamschlacken	2019	2690
6008	red deer	Kamschlacken	2019	5485
6009	red deer	Kamschlacken	2019	820
6014	red deer	Kamschlacken	2019	1002
6011	red deer	Kamschlacken	2019	648
6010	red deer	Kamschlacken	2019	567
6024	red deer	Kamschlacken	2019	929
5117	red deer	Riefensbeek	2019	847
6040	red deer	Kamschlacken	2019	1018
6041	red deer	Kamschlacken	2019	709

4625	red deer	Riefensbeek	2019	1099
5118	red deer	Riefensbeek	2019	982
6015	red deer	Kamschlacken	2019	596
L1056	red deer	Göttingen	May 20	126
L1057	red deer	Göttingen	May 20	100
L1058	red deer	Göttingen	May20	97
130	red deer	Göttingen	Autumn 20	114
131	red deer	Göttingen	Autumn 20	128
132	red deer	Göttingen	Autumn 20	101
133	red deer	Göttingen	Autumn 20	169
249	red deer	Holzminden	Autumn 20	270
250	red deer	Holzminden	Autumn 20	273
251	red deer	Holzminden	Autumn 20	181
252	red deer	Holzminden	Autumn 20	258
253	red deer	Holzminden	Autumn 20	243
254	red deer	Holzminden	Autumn 20	204

Table S9: Overview of tested goat serum samples for ALSV VP2 by LIPS assay.
Red: classified positive, green: classified negative, n=33, positive: 2, Seroprevalence: 6.06%

Sample-ID	Species	Location	Date	VP2-Capsid
SZ64/039	goat	Göttingen	July 17	257
SZ64/041	goat	Göttingen	July 17	152
SZ64/050	goat	Göttingen	July 17	271
SZ64/092	goat	Göttingen	Dec 17	136
SZ64/001	goat	Göttingen	July 18	152
SZ64/013	goat	Göttingen	July 18	737
SZ64/020	goat	Göttingen	July 18	132
SZ64/026	goat	Göttingen	July 18	148
SZ64/027	goat	Göttingen	July 18	174
SZ64/031	goat	Göttingen	July 18	125
SZ64/059	goat	Göttingen	July 18	148
SZ64/060	goat	Göttingen	July 18	195
Z-P17	goat	Hildesheim	January 19	3674
Z-P155	goat	Harz	Febuary 19	286
Z-P165	goat	Peine	Febuary 19	618
Z-P208	goat	Hildesheim	March 19	279
Z-P265	goat	Hildesheim	April 19	227
Z-P272	goat	Hildesheim	April 19	262
Z-P300	goat	Hildesheim	April 19	276
Z-P308	goat	Peine	April 19	350
Z-P365	goat	Peine	April 19	1147
Z-P474	goat	Peine	June 19	304
SZ64/054	goat	Göttingen	July 19	120
SZ64/062	goat	Göttingen	July 19	115

SZ64/117	goat	Göttingen	July 19	86
Z-P545	goat	Hildesheim	July 19	318
SZ64/027	goat	Göttingen	July 20	275
SZ64/055	goat	Göttingen	July 20	190
Z52	goat	Wolfenbüttel	November 20	340
Z57	goat	Northeim	November 20	249
Z68	goat	Celle	November 20	289
Z70	goat	Wolfenbüttel	November 20	308
Z72	goat	Hildesheim	November 20	303

Table S10: Overview of tested sheep serum samples for ALSV VP2 by LIPS assay.
Red: classified positive, green: classified negative, n= 55, positive: 2, seroprevalence: 3.63%.

**Sample-ID**	**Species**	**Location**	**Date**	**VP2-Capsid**
SZ64/004	sheep	Göttingen	July 17	195
SZ64/009	sheep	Göttingen	July 17	211
SZ64/021	sheep	Göttingen	July 17	214
SZ64/026	sheep	Göttingen	December 17	209
SZ64/027	sheep	Göttingen	December 17	182
SZ64/034	sheep	Göttingen	December 17	186
SZ64/024	sheep	Göttingen	July 18	208
SZ64/070	sheep	Göttingen	July 19	203
SZ64/076	sheep	Göttingen	July 19	244
SZ64/080	sheep	Göttingen	July 19	319
SZ64/089	sheep	Göttingen	July 19	204
SZ64/094	sheep	Göttingen	July 19	194
S4	sheep	Hameln	August 19	300
S5	sheep	Hameln	August 19	376
S6	sheep	Diepholz	August 19	520
S7	sheep	Diepholz	August 19	317
S8	sheep	Göttingen	August 19	327
S9	sheep	Göttingen	August 19	412
S16	sheep	Diepholz	August 19	414
S17	sheep	Diepholz	August 19	740
S18	sheep	Diepholz	August 19	724
S31	sheep	Göttingen	August 19	421
S32	sheep	Hameln	August 19	274
S33	sheep	Hameln	August 19	401
S-P294	sheep	Hildesheim	May 19	254
S-P295	sheep	Celle	May 19	289
S-P296	sheep	Celle	May 19	169
S-P324	sheep	Hildesheim	May 19	229
S-P331	sheep	Celle	May 19	245

S-P336	sheep	Celle	May 19	179
S-P343	sheep	Hanover	May 19	208
S-P346	sheep	Hameln	May 19	226
S-P391	sheep	Göttingen	May 19	279
S-P413	sheep	Celle	June 19	315
S-P433	sheep	Braunschweig	June 19	233
S-P453	sheep	Hildesheim	June 19	321
S-P514	sheep	Diepholz	July 19	175
S39	sheep	Diepholz	September 19	295
SZ64/080	sheep	Göttingen	July 20	203
SZ64/104	sheep	Göttingen	July 20	160
S88	sheep	Diepholz	November 20	559
S91	sheep	Wolfenbüttel	November 20	361
S93	sheep	Hildesheim	November 20	332
S94	sheep	Hildesheim	November 20	370
S129	sheep	Nienburg	November 20	303
S133	sheep	Hildesheim	November 20	602
S144	sheep	Diepholz	November 20	1017
S147	sheep	Braunschweig	November 20	288
S148	sheep	Peine	November 20	367
S150	sheep	Wolfsburg	November 20	461
S157	sheep	Hameln	November 20	2463
S158	sheep	Celle	November 20	383
S159	sheep	Celle	November 20	303
S163	sheep	Hildesheim	November 20	276
S165	sheep	Hameln	November 20	1733

Table S11: Overview of tested horse serum samples for ALSV VP2 by LIPS assay.
Red: classified positive, green: classified negative, n=91, positive: 13, seroprevalence: 14.28%.

Sample-ID	Species	Location	Date	VP2-Capsid
P4	horse	Nienburg	Febuary 19	211
P10	horse	Göttingen	Febuary 19	313
P33	horse	Nienburg	Febuary 19	146
P8	horse	Celle	March 19	362
P27	horse	Nienburg	March 19	199
P34	horse	Nienburg	March 19	228
P41	horse	Werningerode	March 19	1024
P48	horse	Hameln	March 19	790
P49	horse	Braunschweig	March 19	584
P50	horse	Celle	March 19	398
P51	horse	Braunschweig	March 19	222
P52	horse	Celle	March 19	439
P60	horse	Hameln	March 19	229
P64	horse	Salzgitter	April 19	172
P66	horse	Northeim	April 19	3077
P68	horse	Peine	April 19	422

P71	horse	Göttingen	April 19	2733
P73	horse	Goslar	May 19	2736
P79	horse	Peine	May 19	1946
P81	horse	Göttingen	May 19	2124
P82	horse	Peine	May 19	182
P85	horse	Peine	May 19	340
P90	horse	Nienburg	May 19	1995
P91	horse	Goslar	May 19	1624
P92	horse	Nienburg	May 19	1318
P93	horse	Holzminden	May 19	1992
P94	horse	Peine	May 19	305
P96	horse	Salzgitter	May 19	245
P98	horse	Salzgitter	May 19	307
P101	horse	Peine	June 19	282
P102	horse	Northeim	June 19	358
P104	horse	Braunschweig	June 19	1438
P105	horse	Celle	June 19	1336
P111	horse	Schaumburg	June 19	1225
P112	horse	Göttingen	June 19	139
P114	horse	Wolfenbüttel	June 19	124
P115	horse	Holzminden	June 19	156
P120	horse	Northeim	June 19	140
P126	horse	Braunschweig	July 19	154
P128	horse	Göttingen	July 19	104
P130	horse	Wolfenbüttel	July 19	410
P131	horse	Holzminden	July 19	214
P132	horse	Celle	July 19	121
P134	horse	Schaumburg	July 19	267
P136	horse	Peine	July 19	164
P138	horse	Göttingen	August 19	111
P140	horse	Wolfenbüttel	August 19	133
P145	horse	Celle	August 19	418
P148	horse	Celle	August 19	232
P161	horse	Hildesheim	August 19	210
P163	horse	Braunschweig	August 19	180
P166	horse	Celle	August 19	114
P168	horse	Braunschweig	August 19	140
P169	horse	Peine	August 19	162
P172	horse	Peine	August 19	84
P175	horse	Wolfenbüttel	August 19	1489
P176	horse	Braunschweig	August 19	175
P182	horse	Nienburg	September 19	144
P185	horse	Harz	September 19	109
P189	horse	Hildesheim	September 19	161

P190	horse	Celle	September 19	252
P191	horse	Hildesheim	September 19	102
P192	horse	Northeim	September 19	151
P218	horse	Nienburg	October 19	289
P224	horse	Nienburg	October 19	229
P269	horse	Nienburg	October 19	281
P284	horse	Göttingen	October 19	442
P274	horse	Celle	October 19	203
P325	horse	Northeim	October 19	279
P313	horse	Goslar	October 19	168
P221	horse	Northeim	October 19	161
P225	horse	Goslar	October 19	188
P226	horse	Goslar	October 19	291
P227	horse	Northeim	October 19	149
P230	horse	Nienburg	October 19	148
P240	horse	Wolfenbüttel	October 19	152
P242	horse	Celle	October 19	154
P254	horse	Göttingen	October 19	145
P256	horse	Salzgitter	October 19	174
P259	horse	Holzminden	October 19	149
P377	horse	Goslar	December 19	229
P411	horse	Harz	January 20	175
P425	horse	Goslar	Febuary 20	214
P434	horse	Nienburg	Febuary 20	377
P451	horse	Harz	March 20	136
P465	horse	Göttingen	March 20	342
P479	horse	Nienburg	April 20	248
P485	horse	Salzgitter	April 20	830
P480	horse	Göttingen	April 20	620
P484	horse	Göttingen	April 20	280
P542	horse	Göttingen	June 20	383

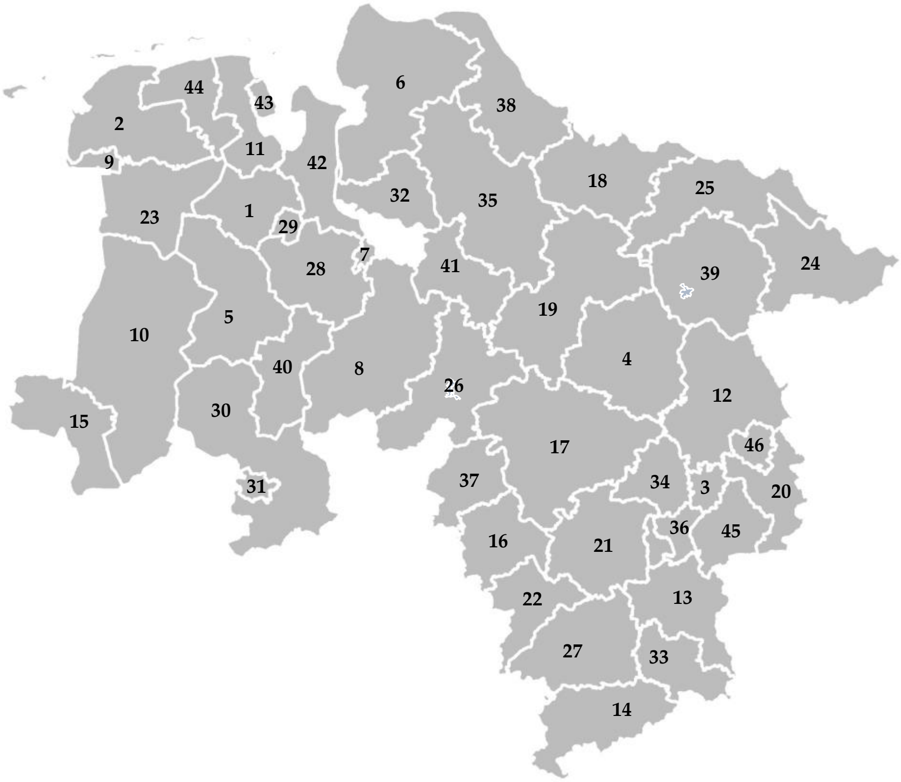

Figure S1: Map of Lower Saxony with Regions. Map was created in R v. 4.1.0 with administrative boundaries retrieved from the Global Administrative Areas Database (gadm.org).

No.	District
1	Ammerland
2	Aurich
3	Braunschweig
4	Celle
5	Cloppenburg
6	Cuxhaven
7	Delmenhorst
8	Diepholz
9	Emden
10	Emsland
11	Friesland
12	Gifhorn
13	Goslar
14	Göttingen
15	Grafschaft Bentheim
16	Hameln-Pyrmont
17	Hanover
18	Harburg
19	Heidekreis
20	Helmstedt
21	Hildesheim
22	Holzminden
23	Leer
24	Lüchow-Dannenberg
25	Lüneburg
26	Nienburg/Weser

27	Northeim
28	Oldenburg
29	Oldenburg
30	Osnabrück
31	Osnabrück
32	Osterholz
33	Osterode am Harz
34	Peine
35	Rotenburg (Wümme)
36	Salzgitter
37	Schaumburg
38	Stade
39	Uelzen
40	Vechta
41	Verden
42	Wesermarsch
43	Wilhelmshaven
44	Wittmund
45	Wolfenbüttel
46	Wolfsburg
